# Optimization and Evaluation of Accelerated Corrosion Tests Based on Mechanism Equivalence Principles

**DOI:** 10.3390/ma17164042

**Published:** 2024-08-14

**Authors:** Mumeng Wei, Jinghua Yao, Yufan Chen, Bojun Yang, Dichun Chen, Yikun Cai

**Affiliations:** 1 State Key Laboratory for Marine Corrosion and Protection, Luoyang 471000, China; syamm2011@163.com (M.W.); yaolele725@163.com (J.Y.); chenyufan96111@hotmail.com (Y.C.); yangbojun.123@163.com (B.Y.); chunzi_79@163.com (D.C.); 2 School of Aeronautics and Astronautics, Sichuan University, Chengdu 610065, China

**Keywords:** indoor corrosion test, corrosion mechanism equivalence, test optimization and design, dynamic link function, experimental methods

## Abstract

Conventional indoor corrosion test design methods primarily focus on the rapid evaluation of material corrosion resistance, often neglecting the impact of environmental stress levels on the equivalence of corrosion mechanisms. This study introduces a novel indoor corrosion test design method based on the principle of corrosion mechanism equivalence, aimed at improving the accuracy of indoor accelerated corrosion simulations. We define the characteristic of corrosion mechanism equivalence as the Corrosion Mechanism Equivalence Degree (CMed), which quantifies the similarity between corrosion mechanisms in indoor accelerated tests and field tests. Then, modified conventional link function models are defined, integrating the probability distribution of environmental factors to estimate corrosion model parameters more precisely. Finally, an optimization problem is constructed for accelerated corrosion tests based on CMed, incorporating constraints on environmental stress levels and acceleration factors. A case study demonstrates the proposed method’s ability to accurately simulate the actual service environment of materials, determining the appropriate stress levels for indoor accelerated corrosion tests while ensuring the desired acceleration factor and corrosion mechanism equivalence.

## 1. Introduction

Field exposure corrosion tests are widely used in corrosion research due to their ability to provide insights into the corrosion behavior of materials in actual service environments [1,2]. However, these tests are often characterized by lengthy durations, high costs, and complexities in developing reliable predictive models. Indoor accelerated corrosion tests, on the other hand, offer a more expedient alternative, delivering extensive information in a significantly shorter time frame and facilitating the prediction of material corrosion failure under normal conditions [3,4,5]. As a result, indoor accelerated corrosion tests have gained considerable attention in the research community.

To design effective indoor accelerated corrosion tests, it is essential to ensure the equivalence of corrosion mechanisms between indoor and field test methods. Several methods have been proposed to evaluate the comparability of corrosion processes, including grey relational analysis [6,7], correlation coefficients [8,9,10], and evaluations based on corrosion morphology and product structure [11,12]. These methods have been applied to assess the comparability of corrosion processes. Grey relational analysis, a commonly used method in metal and coating [8] corrosion tests, involves geometric comparisons of time series data.

However, traditional grey modeling requires equidistant data, which is often not feasible due to unpredictable measurement challenges in corrosion tests [13]. Correlation coefficients, calculated by comparing the slope of performance degradation over time, assume a proportional relationship across different stages, which may not reflect actual engineering scenarios [8]. Qualitative assessments based on corrosion morphology and product structure lack the ability to quantify the equivalence of corrosion mechanisms between indoor and field tests. Thus, there is a need for a novel evaluation method that quantifies corrosion mechanism equivalence while maintaining accuracy even with incomplete data.

To accurately predict the corrosion performance of materials in service environments, it is necessary to design indoor corrosion tests that closely replicate field conditions. Currently, three primary design methods are considered: accelerated corrosion tests based on environmental spectrum [14,15], experimental design [16,17], and statistical fitting [18,19]. Although the environmental spectrum method can quickly assess relative corrosion resistance, it seldom provides quantitative insights into the corrosion process, and standards like ISO 12944 [20] fail to precisely capture the relationship between material corrosion and environmental variations over time [21]. These methods are used to study the corrosion process for both metallic materials and organic coatings [21]. However, this test cannot capture the relationship between material corrosion and changes in environment and time accurately.

Therefore, from this perspective, an accelerated corrosion test based on experiment design shows significant advantages. Its basic approach is to design levels and number of primary environmental factors affecting corrosion to obtain several combinations of acceleration conditions, which are ultimately processed into quantitative acceleration models through experimental data. Researchers have designed indoor corrosion tests for temperature [16,22], humidity [16,23], chloride ion deposition rate [24,25], sulfur dioxide concentration [26,27], etc., and they have established quantitative corrosion models based on test results.

Experimental design methods, which optimize the levels and combinations of key environmental factors influencing corrosion, demonstrate significant advantages but often face challenges in prediction accuracy and precision. Consequently, various optimization criteria based on statistical fitting have been proposed, such as the A-optimal criterion [28], D-optimal criterion [29], and V-optimal criterion [30]. While these criteria mainly focus on the enhancement of prediction accuracy, the equivalence of the corrosion mechanism is not guaranteed, rendering predictions invalid if mechanisms change. Hence, a new optimization criterion primarily considering corrosion mechanism equivalence is imperative.

This study focuses on mechanism-based experimental optimization design for metallic materials and organic corrosion-resistant coatings. We examine the correlation between indoor and field corrosion degradation while maintaining constant corrosion mechanisms based on the acceleration factor invariant principle [31]. We introduce the Corrosion Mechanism Equivalence Degree (CMed) and propose a criterion for evaluating indoor accelerated corrosion tests based on CMed. Additionally, we modify link function models using dynamic environmental factors and historical corrosion data. Finally, we establish an optimization problem for indoor accelerated corrosion tests based on the modified link function model and CMed criterion, comparing different test design schemes under various acceleration factor constraints. Notations are listed in Table 1.

## 2. Methodology

In this section, two degradation models are introduced: the metal corrosion model and coating-aging model. Based on these models, the correlation between degradation parameters in indoor and field corrosion tests are derived using the principle of corrosion mechanism equivalence, thus defining the Corrosion Mechanism Equivalence Degree (*CMed*) to evaluate indoor corrosion tests.

### 2.1. Corrosion Mechanism Equivalence

Comprehensive corrosion failure data from field tests often necessitates protracted testing durations. To expedite this process, researchers frequently employ indoor corrosion tests with accelerated environments, enabling the rapid acquisition of material corrosion data. These indoor corrosion tests are fundamentally accelerated degradation tests, which accelerate the corrosion of materials by intensifying test conditions while preserving the underlying corrosion failure mechanism. This approach facilitates the collection of corrosion data within a shortened time period.

The current analytical frameworks for accelerated test data are predicated on the foundational hypothesis of accelerated experiments proposed by Pieruschka [32]. This hypothesis asserts that in accelerated degradation tests, if the degradation mechanism remains consistent across different stress levels, the degradation trajectory maintains the same form. In essence, variations in stress levels only affect the parameters of the degradation path, not its inherent form. This hypothesis provides a linkage between failure mechanisms and degradation models.

However, in the context of indoor accelerated corrosion tests, the intrinsic relationship among specific model parameters within the degradation model remains ambiguous. Therefore, the acceleration factor invariant principle is introduced to delineate the relationship between model parameters [33]. According to this principle, when the corrosion mechanism remains unchanged, the acceleration corrosion factor is dependent solely on the severity of the corrosion environment, independent from the duration of corrosion exposure.

It is postulated that the corrosion damage incurred by materials in an indoor accelerated corrosion environment $s_{i}$ and field natural environment $s_{f}$ for corrosion times $t_{i}$ and $t_{f}$ are $d_{i}\left(t_{i}\right)$ and $d_{f}\left(t_{f}\right)$, respectively. When $d_{i}\left(t_{i}\right)=d_{f}\left(t_{f}\right)$, the ratio of the two corrosion times is called the acceleration corrosion factor K.

(1) $$K=\frac{t_{f}}{t_{i}}$$

According to the acceleration factor invariant principle, K in the above equation is only related to the corrosion environment factor and is independent from corrosion time. Therefore, for any field environmental corrosion time $t_{f}$, there must be a corresponding indoor accelerated corrosion time $t_{i}$ that satisfies the following equation.

(2) $$d_{f}\left(Kt_{i}\right)=d_{i}\left(t_{i}\right)$$

The term containing $t_{i}$ in the expression of K derived from the above equation should be eliminated to satisfy the acceleration factor invariant principle. Based on this, a quantitative relationship between the corrosion degradation model parameters when the indoor accelerated corrosion test is equivalent to the field environmental corrosion mechanism can be obtained.

The acceleration factor invariant principle is the mathematical criterion of mechanism equivalence. In practical applications, we should first verify the consistency of the degradation mechanism; then, the acceleration factor invariant principle is used to determine model parameters.

### 2.2. Metal Corrosion Test Evaluation Criterion

The atmospheric corrosion law of metal materials can usually be described by the following [34,35].

(3) $$C\left(t\right)=At^{n}$$

where C represents corrosion loss, such as corrosion loss, corrosion depth, etc.; A and n are parameters of the corrosion model; and t is corrosion time. Using this formula as the corrosion degradation function of the metal material, the corrosion loss of in the field environment can be expressed as

(4) $$C_{f}\left(t_{f}\right)=A_{f}{t_{f}}^{n_{f}}$$

where $C_{f}$ represents the corrosion loss from field exposure tests, and $A_{f}$ and $n_{f}$ are parameters of the field corrosion model, with $t_{f}$ denoting the duration of the exposure. Similarly, the corrosion loss from indoor accelerated tests can be expressed as follows.

(5) $$C_{i}\left(t_{i}\right)=A_{i}{t_{i}}^{n_{i}}$$

where $C_{i}$ represents the corrosion loss from laboratory accelerated tests, and $A_{i}$ and $n_{i}$ are parameters of the indoor corrosion model, with $t_{i}$ denoting the duration of the exposure. By setting $C_{i}\left(t_{i}\right)=C_{f}\left(t_{f}\right)$, it can be obtained from Equation (2) that

(6) $$A_{i}{t_{i}}^{n_{i}}=A_{f}{\left(Kt_{i}\right)}^{n_{f}}$$

From the above equation, it can be derived that

(7) $$K^{n_{f}}=\frac{A_{i}}{A_{f}}\cdot \frac{{t_{i}}^{n_{i}}}{{t_{i}}^{n_{f}}}$$

According to the acceleration factor invariant principle, the term in Equation (7) that is dependent on $t_{i}$ should be eliminated. Hence, the condition of corrosion mechanism equivalence between indoor and field corrosion tests can be derived as follows.

(8) $$n_{i}=n_{f}$$

Based on Equation (8), the expression for the acceleration factor is

(9) $$K={\left(\frac{A_{i}}{A_{f}}\right)}^{\frac{1}{n_{f}}}$$

The condition for equivalence of failure mechanisms between indoor and field corrosion tests, as derived from the assumed corrosion degradation model for metallic materials, implies that when the failure mechanism remains unchanged, the corrosion degradation model parameters for the two corrosion environments must satisfy the relationship expressed in Equation (8). In order to facilitate the assessment of the equivalence of corrosion mechanisms in indoor and field metal corrosion tests, the *CMed* in the metal corrosion model is defined as the relative difference between the parameters that should remain consistent between the two corrosion environments.

(10) $$CMed=1-\left|\frac{n_{i}-n_{f}}{n_{f}}\right|$$

The closer the *CMed* is to 1, the more similar the corrosion mechanisms in indoor and field metal corrosion tests are, indicating a better simulation effect of the indoor test.

### 2.3. Coating-Aging Test Evaluation Criterion

Coating-aging degradation models have multiple forms due to different observation indicators. In this paper, the two most widely used evaluation indicators in the field of coating evaluation (gloss loss and electrochemical impedance) are selected to establish the evaluation criteria for coating-aging tests.

In 2003, Guseva proposed using the gloss at an incident angle of 60° as an indicator of coating aging [27]. And the model describing the degradation of coating gloss can refer to Ref. [36], where an organic coating gloss loss degradation model is derived from the results of coating-aging tests. This model can be expressed as follows.

(11) $$\Delta G\left(t\right)=\eta \cdot \left[\exp\left(\lambda t\right)-1\right]$$

(12) $$\Delta G\left(t\right)=\frac{G_{0}-G\left(t\right)}{G_{0}}$$

where ΔG represents the gloss loss of the coating, $G_{0}$ represents the initial gloss of the coating, $G\left(t\right)$ represents the gloss of the coating at time t, and η and λ are parameters of the coating gloss-aging model.

Using Equation (11) as the gloss degradation model for organic coatings, the gloss loss in the field environment can be expressed as follows.

(13) $${\Delta G}_{f}\left(t_{f}\right)=\eta_{f}\cdot \left[\exp\left(\lambda_{f}t_{f}\right)-1\right]$$

where ${\Delta G}_{f}$ represents the gloss loss from field coating-aging tests, and $\eta_{f}$ and $\lambda_{f}$ are parameters of the field gloss degradation model, with $t_{f}$ denoting the duration of the field aging test. Similarly, the gloss loss from indoor accelerated coating-aging tests can be expressed as follows.

(14) $${\Delta G}_{i}\left(t_{i}\right)=\eta_{i}\cdot \left[\exp\left(\lambda_{i}t_{i}\right)-1\right]$$

where ${\Delta G}_{i}$ represents the gloss loss from indoor accelerated coating-aging tests, and $\eta_{i}$ and $\lambda_{i}$ are parameters of the indoor gloss aging model, with $t_{i}$ denoting the duration of the indoor aging test. By setting ${\Delta G}_{i}={\Delta G}_{j}$, as per Equation (2), it follows that

(15) $$\eta_{i}\cdot \left[\exp\left(\lambda_{i}t_{i}\right)-1\right]=\eta_{f}\cdot \left[\exp\left(\lambda_{f}{Kt}_{i}\right)-1\right]$$

According to the acceleration factor invariant principle, the term in Equation (15) that is dependent on $t_{i}$ should be eliminated. Hence, the condition for equivalence of mechanisms in indoor and field organic coating gloss-aging tests can be derived as follows.

(16) $$\eta_{i}=\eta_{f}$$

Based on Equation (16), the expression for the acceleration factor of coating gloss aging is obtained as follows.

(17) $$K=\frac{\lambda_{i}}{\lambda_{f}}$$

The corrosion mechanism equivalence condition, derived from the assumed gloss degradation model for organic coatings, indicates that the parameters of the gloss degradation model for indoor and field organic coating gloss-aging tests must satisfy Equation (16) when the corrosion failure mechanism remains constant. To evaluate the equivalence of corrosion mechanisms in indoor and field organic coating gloss-aging tests, the CMed in the organic coating gloss degradation model is defined as the relative difference between the parameters that should remain consistent between the two corrosion environments.

(18) $$CMed=1-\left|\frac{\eta_{i}-\eta_{f}}{\eta_{f}}\right|$$

In addition to gloss, coating electrochemical impedance is also a widely used performance evaluation indicator for coatings. Bierwagen analyzed the results of aging tests for various organic coatings and proposed a coating-aging kinetics model, which describes the degradation of coating impedance using an exponential function [37].

(19) $$Z\left(t\right)=\alpha \cdot \exp\left(\beta t\right)$$

where Z represents the impedance modulus of the coating at low frequencies, while α and β are parameters of the coating impedance degradation model. 

Using the aging kinetics model as the impedance degradation model for organic coatings, the degradation of coating impedance in a field environment can be expressed as follows.

(20) $$Z_{f}\left(t_{f}\right)=\alpha_{f}\cdot \exp\left(\beta_{f}t_{f}\right)$$

where $Z_{f}$ represents the low-frequency impedance modulus from field coating-aging tests, while $\alpha_{f}$ and $\beta_{f}$ are parameters of the field impedance degradation model, with $t_{f}$ denoting the duration of the field aging test. Similarly, the impedance degradation model for indoor accelerated coating-aging tests can be expressed as follows.

(21) $$Z_{i}\left(t_{i}\right)=\alpha_{i}\cdot \exp\left(\beta_{i}t_{i}\right)$$

where $Z_{i}$ represents the low-frequency impedance modulus from indoor accelerated coating-aging tests, and $\alpha_{i}$ and $\beta_{i}$ are parameters of the indoor impedance-aging model, with $t_{i}$ denoting the duration of the indoor aging test. By setting $Z_{j}\left(t_{j}\right)=Z_{i}\left(t_{i}\right)$, as derived from Equation (2), it can be observed that

(22) $$\alpha_{i}\cdot \exp\left(\beta_{i}t_{i}\right)=\alpha_{f}\cdot \exp\left(\beta_{f}{Kt}_{i}\right)$$

Equation (22) can be rearranged as

(23) $$K=\frac{1}{{t_{i}\beta }_{f}}ln\left(\frac{\alpha_{i}}{\alpha_{f}}\right)+\frac{\beta_{i}}{\beta_{f}}$$

According to the acceleration factor invariant principle, the term in Equation (23) that is dependent on $t_{i}$ should be eliminated. Hence, the condition for equivalence of mechanisms in indoor and field organic coating impedance degradation tests can be derived as follows.

(24) $$\alpha_{i}=\alpha_{f}$$

Based on Equation (24), the expression for the acceleration factor of coating impedance degradation can be derived as

(25) $$K=\frac{\beta_{i}}{\beta_{f}}$$

Under the assumption of the organic coating impedance degradation model, the condition for equivalence of corrosion mechanisms in indoor and field organic coating impedance degradation tests indicates that the parameters of the impedance degradation model for the two corrosion environments must satisfy the relationship expressed in Equation (24) when the failure mechanism remains unchanged. To evaluate the equivalence of the corrosion mechanism in indoor and field organic coating impedance degradation, the *CMed* in the coating impedance degradation model is defined as the relative difference between the parameters that should remain consistent between the two corrosion environments.

(26) $$CMed=1-\left|\frac{\alpha_{i}-\alpha_{f}}{\alpha_{f}}\right|$$

### 2.4. Validation of the Evaluation Criterion

In this section, two cases are studied to illustrate the process of determining and verifying the proposed CMed-based evaluation criterion.

- **Case I**: indoor and field corrosion test of 7B04 aluminum alloy

In this case, we conducted two field natural exposure tests and three indoor simulated corrosion tests on 7B04 aluminum alloy to investigate the equivalence of different simulated corrosion environments with the field environments. To begin, 7B04 aluminum alloy was selected as the material for the test specimens, and its chemical composition is shown in Table 2. The dimensions of the corrosion specimens are 100 mm × 50 mm × 6.5 mm, with three parallel specimens tested for each condition. Prior to the tests, the specimens are cleaned with acetone to remove surface oil, rinsed with distilled water, and dried before weighing.

Two field natural exposure tests are conducted at the Jiangjin test station in Chongqing, China, and the Wanning test station in Hainan, China. The Jiangjin region is a typical sub-humid and hot acid rain atmospheric environment with high temperatures, high humidity, and frequent acid rain. Wanning is in a typical tropical marine atmospheric environment characterized by high temperatures, high humidity, and a high deposition rate of chloride ions. The average atmospheric environment observation data for the two test stations are shown in Table 3.

The conditions for the three types of indoor corrosion tests are as follows:

(1) Salt spray test: This test is conducted in accordance with the ASTM B117-97 standard [38]. The test solution is 5.0% NaCl, with the pH adjusted to 6.7–7.2 using 1% HCl and 1% NaOH solution, and the temperature in the test chamber maintained at 35 °C;
(2) Cyclic immersion test: This test is conducted in reference to the HB 5194-1981 standard [39]. The test solution is 5% NaCl solution + 0.8% Na_2_S_2_O_8_ solution + 0.05% (NH_4_)_2_SO_4_ solution, with the pH adjusted to 3.8–4.0 using 10% glacial acetic acid. The test is conducted using a cycle of immersion and drying, with immersion conditions of 35 °C for 10 min and drying conditions of 35 °C air temperature and 75% relative humidity for 50 min;
(3) SO_2_/salt spray combined cyclic test: This test is not based on a standard and is independently designed by our research team. It involved a cyclic test of salt spray, SO_2_ gas injection, and immersion, with a salt spray phase of 30 min, SO_2_ gas injection phase of 30 min, and immersion phase of 120 min, conducted in that sequence. The test parameters for the cyclic test are shown in Table 4.

**Figure 1:**
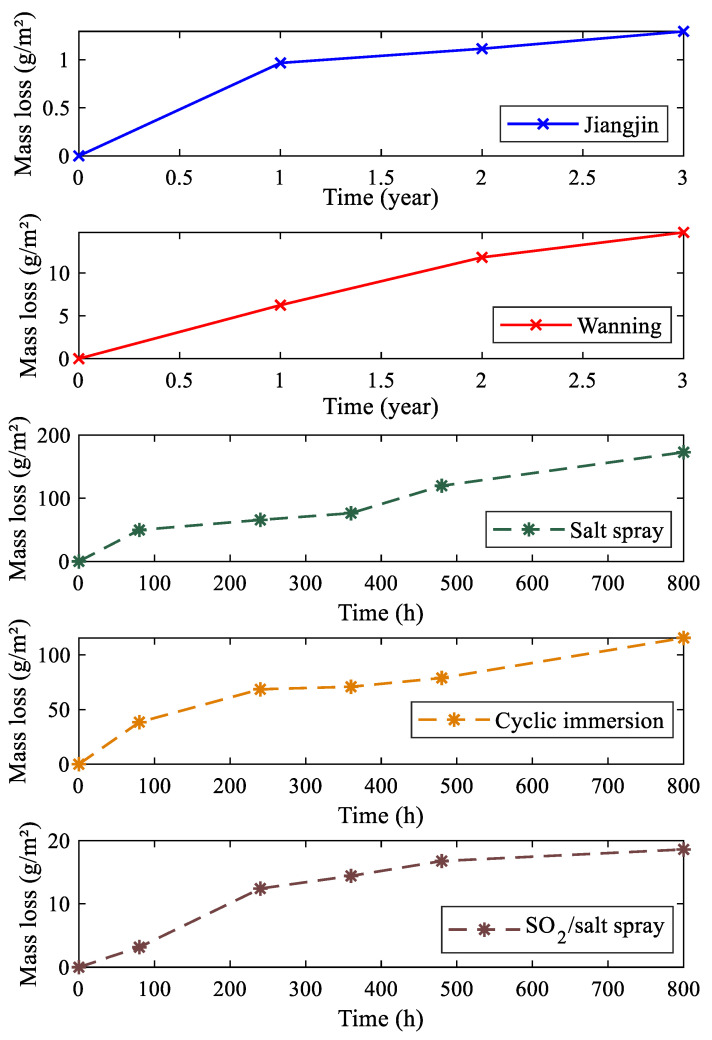
Mass loss data from different corrosion tests.

For the field exposure tests, samples are taken after 1, 2, and 3 years, while for the indoor tests, samples are collected after 80, 240, 360, 480, and 800 h. Following sampling, the corrosion products are cleared in accordance with the HB 5257-1983 standard [40], and the samples are dehydrated using alcohol, dried, and left to stand in a dryer for 24 h before being weighed, and then the weight change is recorded. The test results are presented in Figure 1.

The mass loss data in Figure 1 are fitted using Equation (3) to obtain the corrosion degradation model parameters for different corrosion environments, which are presented in Table 4. The goodness-of-fit for the 5 data sets indicates that the corrosion degradation models have a satisfactory fitting effect, confirming the validity of the corrosion kinetics model. The time units for the field test data and indoor test data in Table 4 are different, but they do not affect the calculation of CMed. Specifically, the different time units only affect the estimation results of parameter A and not parameter n.

Based on the parameter estimation results in Table 5, CMed for indoor simulated corrosion environments and the field environments are calculated using Equation (10), and the results are presented in Table 6. It can be observed that the CMed for the 3 indoor simulated environments and the Jiangjin test station environment are relatively small, indicating that the equivalence between these indoor tests and the Jiangjin field environment is weak. Therefore, the acceleration of corrosion under equivalent corrosion mechanisms has not been achieved, and the actual corrosion situation in the field cannot be reflected.

On the other hand, the CMed for indoor simulated environments and the Wanning test station environment are generally larger than those of Jiangjin, especially for the SO_2_/salt spray combined cyclic test that we designed independently, which has a CMed of 0.9994 for the Wanning test station environment. The result indicates that the SO_2_/salt spray combined cyclic test can effectively simulate the Wanning test station corrosion environment while ensuring corrosion mechanism equivalence.

- **Case II**: indoor and field corrosion test of polyurethane coating

**Figure 2:**
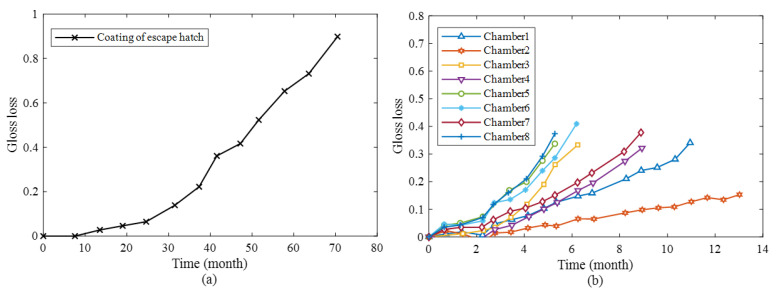
Gloss curves for the polyurethane coating for (**a**) coating of escape hatch and (**b**) indoor test chambers 1–8.

The gloss degradation data of the polyurethane coating in this case study are obtained from Ref. [27]. The study conducted 8 degradation tests of polyurethane coatings in simulated indoor corrosion environments and compared them with the degradation of coatings in the escape hatch of the B-747 in the field. The simulated indoor corrosion environments considered factors such as temperature, UV radiation, and sulfur dioxide aerosols, with sulfur dioxide aerosols only used as a comparative factor. The simulated indoor environmental conditions are listed in Table 6, and the gloss degradation data of the field coatings and the simulated indoor degradation data are presented in Figure 2. Detailed experimental procedures can be found in Ref. [27].

We fit the gloss degradation model to the data presented in Figure 2 and use the estimated model parameters to calculate the CMeds for different indoor accelerated coating-aging environments. The parameter estimation results and the CMeds are presented in Table 7 and Table 8, respectively. The high goodness-of-fit of the gloss data under different environments indicates that the model proposed by Wu [36] is appropriate for describing the gloss degradation of polyurethane coatings. Based on the CMeds from different chambers, Chamber 7 has the highest value, followed by Chamber 5. This suggests that under the conditions of temperature around 329 K and UV around 0.63 W/m^2^nm, the equivalence between the indoor coating test and field data is optimal in terms of the observation index of gloss. It is possible that sulfur dioxide aerosols do not alter the equivalence of indoor experiments, but only affect the acceleration factor.

Actually, the proposed CMed method is quite flexible to be used in different situations. The most important is to find the parameter that can describe the process of corrosion degradation. When uniformity happens, corrosion mass loss or depth is the suitable parameter. When pitting occurs, pit depth or density would be the suitable parameter, while for coatings, electrochemical parameters (such as low frequency impedance, characteristic frequency, etc.) would be the right ones.

## 3. Regression Models

Before optimizing the condition design of indoor accelerated testing, it is necessary to establish the relationship between the environmental factors and the corrosion degradation model parameters, i.e., the link function model, to determine the constraints for optimization. There are studies that give a broad view of link functions [41,42]. This section provides three link function models that can be used to describe environmental effects and proposes a time-varying link function that considers dynamic environments.

### 3.1. Two-Step Regression Model

The corrosion behavior of materials is influenced not only by natural environmental factors but also by the progression of time. Typically, the corrosion degradation models discussed in Section 2.1 are used to characterize the temporal evolution of corrosion. Additionally, environmental variables such as temperature, humidity, ultraviolet radiation, and contaminant deposition play a critical role in the corrosion process. Thus, the corrosion degradation process has a layered structure: the first layer captures the time-dependent variation in corrosion, while the second layer accounts for the impact of environmental factors on the degradation parameters. A straightforward method to analyze this multi-layered structure is the two-step regression (TR) approach. First, regression analysis is conducted to examine the temporal progression of corrosion loss, followed by regression analysis to explore the relationship between environmental factors and the parameters derived from the first step. 

The metal corrosion kinetics model and the coating impedance-aging kinetics model presented in Section 2.1 can be linearly transformed to facilitate the first-layer regression models [43].

(27) $$lnC\left(t\right)=lnA+nlnt$$

(28) $$lnZ\left(t\right)=ln\alpha +\beta t$$

The second-layer regression model establishes the relationship between the parameters from the first-layer regression and the environmental variables. For metal corrosion, the relevant environmental factors include temperature, humidity, and chloride ion deposition rate. For the coating impedance-aging model, temperature, ultraviolet radiation, and contaminant deposition are considered. Thus, the second-layer model can be expressed as

(29) $$\left\{\begin{array}{c} A=\vec{\gamma_{A}}\cdot \vec{E_{m}}\\ n=\vec{\gamma_{n}}\cdot \vec{E_{m}} \end{array}\right.$$

(30) $$\left\{\begin{array}{c} \alpha =\vec{\gamma_{\alpha }}\cdot \vec{E_{c}}\\ \beta =\vec{\gamma_{\beta }}\cdot \vec{E_{c}} \end{array}\right.$$

where $\vec{E_{m}}$ and $\vec{E_{c}}$ are the vectors of average environmental factors affecting metal corrosion and coating impedance aging, respectively. $\vec{E_{m}}={\left[\frac{1}{\bar{T}+273},\;\bar{RH},\bar{Cl}\right]}^{T},\;and\;\;\vec{E_{c}}={\left[\frac{1}{\bar{T}+273},\;\bar{UV},\bar{Cl}\right]}^{T}$. $\vec{\gamma_{A}}$, $\vec{\gamma_{n}}$, $\vec{\gamma_{\alpha }}$, and $\vec{\gamma_{\beta }}$ are the coefficient vectors of environmental factors regarding parameters A, n, α, and β, respectively. $\vec{\gamma_{A}}=\left[\gamma_{A0},\gamma_{A1},\gamma_{A2},\gamma_{A3}\;\right]$, $\vec{\gamma_{n}}=\left[\gamma_{n0},\gamma_{n1},\gamma_{n2},\gamma_{n3}\;\right]$, $\vec{\gamma_{\alpha }}=\left[\gamma_{\alpha 0},\gamma_{\alpha 1},\gamma_{\alpha 2},\gamma_{\alpha 3}\;\right]$, and $\vec{\gamma_{\beta }}=\left[\gamma_{\beta 0},\gamma_{\beta 1},\gamma_{\beta 2},\gamma_{\beta 3}\;\right]$.

For the coating gloss degradation model, linear transformation is not applicable, and the model parameters can be obtained through the nonlinear least squares method, and then the second-layer regression model can be established.

(31) $$\left\{\begin{array}{c} \eta =\vec{\gamma_{\eta }}\cdot \vec{E_{c}}\\ \lambda =\vec{\gamma_{\lambda }}\cdot \vec{E_{c}} \end{array}\right.$$

where $\vec{\gamma_{\eta }}$ and $\vec{\gamma_{\lambda }}$ are the coefficient vectors of environmental factors about parameter η and λ, respectively. $\vec{\gamma_{\eta }}=\left[\gamma_{\eta 0},\gamma_{\eta 1},\gamma_{\eta 2},\gamma_{\eta 3}\right]$, and $\vec{\gamma_{\lambda }}=\left[\gamma_{\lambda 0},\gamma_{\lambda 1},\gamma_{\lambda 2},\gamma_{\lambda 3}\right].$

The two-step regression model is relatively straightforward and computationally efficient. However, due to the potential nonlinear relationships between environmental factors and model parameters, the accuracy of the two-step regression model may be limited.

### 3.2. General Eyring Model

The General Eyring (GE) model is a model used to describe the degradation behavior of materials under the influence of multiple environmental factors. Existing multi-stress coupling models are mainly divided into two categories: those based on failure physics and those based on empirical learning. The GE model is based on failure physics, which typically consider the microstructure and chemical composition of materials, as well as the mechanisms by which environmental stress affects materials. The relationship between environmental stress and the actual failure mechanism is sometimes extremely complex, or the failure mechanism is not yet clear. 

In the field of metal corrosion prediction, there are already mature failure physics models. Therefore, for the degradation of metal corrosion, this paper adopts the GE model to establish the relationship between degradation model parameters and environmental factors [42]. When considering environmental factors such as temperature, humidity, and chloride ion deposition rate, the temperature stress model is captured by the Arrhenius equation, the humidity model is captured by the Peck equation, and the chloride ion deposition model is captured by the power function form. Without considering the interaction effect between environmental stresses, the GE model of the metal corrosion degradation process can be expressed as

(32) $$\left\{\begin{array}{c} A=\gamma_{A0}\cdot \exp\left(\frac{-\gamma_{A1}}{\bar{T}+273}\right)\cdot {\bar{RH}}^{\gamma_{A2}}\cdot {\bar{Cl}}^{\gamma_{A3}}\\ n=\gamma_{n0}\cdot \exp\left(\frac{-\gamma_{n1}}{\bar{T}+273}\right)\cdot {\bar{RH}}^{\gamma_{n2}}\cdot {\bar{Cl}}^{\gamma_{n3}}\; \end{array}\right.$$

The failure mechanism of the coating is still unclear, and there are few failure physics models of coating. Therefore, the GE model of coating in this paper mainly refers to the three-stress model proposed by Ref. [27]. When considering environmental factors such as temperature, UV, and sulfur dioxide aerosols, the model can be expressed as follows.

For metal corrosion model:

(33) $$\left\{\begin{array}{c} \eta =\gamma_{\eta 0}\cdot \exp\left(\frac{-\gamma_{\eta 1}}{\bar{T}+273}\right)\cdot {\bar{RH}}^{\gamma_{\eta 2}}\cdot \exp\left(\gamma_{\eta 3}\bar{SO_{2}}\right)\\ \lambda =\gamma_{\lambda 0}\cdot \exp\left(\frac{-\gamma_{\lambda 1}}{\bar{T}+273}\right)\cdot {\bar{RH}}^{\gamma_{\lambda 2}}\cdot \exp\left(\gamma_{\lambda 3}\bar{SO_{2}}\right)\; \end{array}\right.$$

For coating impedance-aging model:

(34) $$\left\{\begin{array}{c} \alpha =\gamma_{\alpha 0}\cdot \exp\left(\frac{-\gamma_{\alpha 1}}{\bar{T}+273}\right)\cdot {\bar{RH}}^{\gamma_{\alpha 2}}\cdot \exp\left(\gamma_{\alpha 3}\bar{SO_{2}}\right)\\ \beta =\gamma_{\beta 0}\cdot \exp\left(\frac{-\gamma_{\beta 1}}{\bar{T}+273}\right)\cdot {\bar{RH}}^{\gamma_{\beta 2}}\cdot \exp\left(\gamma_{\beta 3}\bar{SO_{2}}\right)\; \end{array}\right.$$

### 3.3. General Log-Linear Model

The relationship between environmental stress and actual failure mechanisms can be extremely complex at times, or there may be insufficient understanding of the failure mechanisms. In such cases, an accelerated model based on empirical learning can be used, such as the generalized linear logarithmic model. The GL model is a type of universal model, with the main difference from the GE model being that the form of the temperature acceleration model is different. The GE model assumes that the temperature acceleration model follows either the Eyring model or the Arrhenius model, while the GL model assumes a simple exponential model. In the absence of consideration of interactions between various environmental stresses, its form is expressed as follows [27].

For coating gloss-aging model:

(35) $$\left\{\begin{array}{c} \ln A=\vec{\gamma_{A}}\cdot \vec{E_{m}}\\ \ln n=\vec{\gamma_{n}}\cdot \vec{E_{m}} \end{array}\right.$$

For coating gloss-aging model:

(36) $$\left\{\begin{array}{c} \ln\alpha =\vec{\gamma_{\alpha }}\cdot \vec{E_{c}}\\ \ln\beta =\vec{\gamma_{\beta }}\cdot \vec{E_{c}} \end{array}\right.$$

For coating impedance-aging model:

(37) $$\left\{\begin{array}{c} \ln\eta =\vec{\gamma_{\eta }}\cdot \vec{E_{c}}\\ \ln\lambda =\vec{\gamma_{\lambda }}\cdot \vec{E_{c}} \end{array}\right.$$

When it is necessary to consider the interactions between different environmental stresses, the product of the different environmental stresses can be introduced as a new variable in the GL model.

### 3.4. Dynamic Link Function Model

In the above three conventional link functions, the corrosion environment is simply described by the average value of each environmental factor. However, in the corrosion environment, environmental factors vary continuously and are distributed within a certain range. Predicting the corrosion law by taking the average value of environmental factors over the entire corrosion time not only affects the accuracy of the link function model, but also leads to inaccurate corrosion prediction. Therefore, this paper fully considers the dynamic and stochastic nature of field corrosion environmental factors and uses a probability model to improve the conventional link function model to reflect its variable characteristics.

(38) $$f\left(\vec{E}\right)=\sum_{i=1}^{m}\omega_{i}\cdot f_{i}\left(\vec{E}\right)$$

where $\vec{E}$ represents the fitted environmental factors, m is the number of distributions, and $f_{i}\left(\vec{E}\right)$ is the probability density function of the i-th distribution of environmental factor $\vec{E}$. When the environmental factor distribution is symmetric, $f_{i}\left(\vec{E}\right)$ can be chosen in the form of a normal distribution, and when the environmental factor is asymmetric, $f_{i}\left(\vec{E}\right)$ can be chosen in the form of a lognormal distribution or a Weibull distribution, where $\omega_{i}$ is the weight coefficient of $f_{i}\left(\vec{E}\right)$.

Next, the optimal link function $\xi_{\theta }\left(\vec{E}\right)$ is selected to describe the influence of environmental factors on parameter θ based on the fitting effect of the three traditional link functions. Then, the link function $\xi_{\theta }\left(\vec{E}\right)$ is modified according to the distribution function of the environmental factors, thereby reflecting the dynamic and stochastic nature of the environmental factors. The modified link function can be expressed as

(39) $${\xi^{\prime }}_{\theta }\left(\vec{E}\right)=\int_{{\vec{E}}_{L}}^{{\vec{E}}_{U}}\xi_{\theta }\left(\vec{E}\right)\cdot f\left(\vec{E}\right)dE$$

where ${\vec{E}}_{U}$ represents the upper limit of the environmental factor, and ${\vec{E}}_{L}$ represents the lower limit of the environmental factor.

## 4. Optimization Framework

This section focuses on the optimization design of an indoor corrosion test based on CMed. Specifically, the optimal design of the indoor corrosion test requires optimization of the environmental stress level applied to the indoor corrosion environment based on the principle of equivalent corrosion mechanism under the given acceleration factor and stress level boundary constraints.

### 4.1. Data Collection and Degradation Modeling

The primary objective of indoor corrosion testing is to evaluate or predict the degradation behavior of materials under real-world environmental conditions. Therefore, before designing indoor tests, it is essential to analyze the corrosion mechanisms occurring in the field environment. The potential corrosion mechanisms should be identified based on the composition, application, and service conditions of the materials being tested. This identification process can be informed by literature review, field observations, and expert consultations. For metallic materials, corrosion kinetics models as discussed in Section 2.2 can be employed, while for organic coatings, either gloss degradation models or impedance-aging kinetics models outlined in Section 2.3 can be used, depending on the chosen evaluation metrics.

Next, key environmental factors that significantly influence the corrosion process should be identified based on the actual service environment of the materials. These factors must have a substantial impact on the corrosion behavior and be prevalent in the service conditions. Suppose the number of key environmental factors is h, and then the key environmental factors are represented as ${{\vec{S}=S}_{1},\;S}_{2},\;\ldots ,\;S_{h}$. In addition, it is necessary to collect observational data of these environmental factors in the field, assuming that the average levels of the key environmental factors in the field environment are $\vec{S^{\prime }}={S_{1}}^{\prime },\;S_{2}^{\prime },\;\ldots ,\;S_{h}^{\prime }$.

Following the acquisition of basic field environmental data, a pre-compliance test is necessary. This involves applying multiple levels of environmental stress to the materials and observing their degradation patterns. The stress levels in the pre-compliance test should closely resemble those in the field environment to minimize the risk of altering the corrosion mechanism. The degradation model parameters should then be estimated based on the pre-compliance test results, and a link function should be constructed. The link function can be determined by fitting the degradation data from the pre-compliance test using the link functions described in Section 3.1, Section 3.2 and Section 3.3, selecting the model with the highest goodness-of-fit or the lowest prediction error. For the metal corrosion degradation model, the general form of the link function is

$$A=\xi_{A}\left(\vec{S}\right),n=\xi_{n}\left(\vec{S}\right)$$

where $\xi_{A}\left(\cdot \right)$ represents the link function between parameter A and environmental factors, and $\xi_{n}\left(\cdot \right)$ represents the connection function between parameter n and environmental factors. Therefore, the parameters of the field metal degradation model can be directly calculated as

$$A_{f}=\xi_{A}\left(\vec{S^{\prime }}\right),n_{f}=\xi_{n}\left(\vec{S^{\prime }}\right)$$

Similarly, the expressions for the outdoor degradation model parameters of the coating degradation model can be directly obtained.

For the gloss degradation model:

$$\eta_{f}=\xi_{\eta }\left(\vec{S^{\prime }}\right),\lambda_{f}=\xi_{\lambda }\left(\vec{S^{\prime }}\right)$$

where $\xi_{\eta }\left(\cdot \right)$ represents the link function between parameter η and environmental factors, and $\xi_{\lambda }\left(\cdot \right)$ represents the link function between parameter λ and environmental factors.

For the impedance-aging kinetics model:

$$\alpha_{f}=\xi_{\alpha }\left(\vec{S^{\prime }}\right),\beta_{f}=\xi_{\beta }\left(\vec{S^{\prime }}\right)$$

where $\xi_{\alpha }\left(\cdot \right)$ represents the link function between parameter α and environmental factors, and $\xi_{\beta }\left(\cdot \right)$ represents the connection function between parameter β and environmental factors.

### 4.2. Formulating the Optimization Problem

In indoor corrosion testing, controlling the acceleration factor to keep the testing duration within a reasonable range is crucial for cost efficiency. Thus, the acceleration factor (*K*) should be used as a constraint in the optimization problem. Taking indoor metal corrosion testing as an example, the acceleration factor (*K*) can be expressed as

(40) $$K={\left(\frac{A_{i}}{A_{f}}\right)}^{\frac{1}{n_{f}}}={\left(\frac{\xi_{A}\left(\vec{S}\right)}{\xi_{A}\left(\vec{S^{\prime }}\right)}\right)}^{\frac{1}{\xi_{n}\left(\vec{S^{\prime }}\right)}}$$

Similarly, for indoor coating-aging tests, the acceleration factor (*K*) can be represented as

(41) $$K=\frac{\lambda_{i}}{\lambda_{f}}=\frac{\xi_{\lambda }\left(\vec{S}\right)}{\xi_{\lambda }\left(\vec{S^{\prime }}\right)}$$

or

(42) $$K=\frac{\beta_{i}}{\beta_{f}}=\frac{\xi_{\beta }\left(\vec{S}\right)}{\xi_{\beta }\left(\vec{S^{\prime }}\right)}$$

In addition to the acceleration factor, it is vital to consider the maximum and minimum levels of environmental stress that can be applied using laboratory equipment to avoid altering the corrosion mechanism and ensure test efficiency. The maximum stress level should not exceed the upper limit of stress, ${S_{1}^{H},\;S}_{2}^{H},\;\ldots ,\;S_{h}^{H}$, and the minimum stress level should not be lower than the lower limit of stress, ${S_{1}^{L},\;S}_{2}^{L},\;\ldots ,\;S_{h}^{L}$. With these constraints in place, the optimal indoor corrosion testing scheme, which maximizes the equivalence of the corrosion mechanism, can be established by optimizing the levels of environmental stress. The objective function for optimizing the indoor corrosion test scheme is

(43) $${\left(n_{i}-n_{f}\right)}^{2}={\left(\xi_{n}\left(\vec{S}\right)-\xi_{n}\left(\vec{S^{\prime }}\right)\right)}^{2}$$

Similarly, the objective function for coating-aging test optimization are

(44) $${\left(\eta_{i}-\eta_{f}\right)}^{2}={\left(\xi_{\eta }\left(\vec{S}\right)-\xi_{\eta }\left(\vec{S^{\prime }}\right)\right)}^{2}$$

or

(45) $${\left(\alpha_{i}-\alpha_{f}\right)}^{2}={\left(\xi_{\alpha }\left(\vec{S}\right)-\xi_{\alpha }\left(\vec{S^{\prime }}\right)\right)}^{2}$$

The optimization problem for indoor metal corrosion testing based on CMed can be summarized as follows
Minimize$$\Psi \left(\zeta \right)={\left(\xi_{n}\left(\vec{S}\right)-\xi_{n}\left(\vec{S^{\prime }}\right)\right)}^{2}$$subject to$${\left(\frac{\xi_{A}\left(\vec{S}\right)}{\xi_{A}\left(\vec{S^{\prime }}\right)}\right)}^{\frac{1}{\xi_{n}\left(\vec{S^{\prime }}\right)}}=k$$$${S_{1}^{L}\leq S}_{1}\leq S_{1}^{H}$$$${S_{2}^{L}\leq S}_{2}\leq S_{2}^{H}$$⋯⋯$${S_{h}^{L}\leq S}_{h}\leq S_{h}^{H}$$
where $\Psi \left(\cdot \right)$ represents objective function, $\zeta =\left[S_{1},S_{2},\ldots ,S_{h}\right]$ represents indoor test condition, and k represents the given acceleration factor. The optimization problem for the indoor coating aging test is similar.

Finally, the optimal environmental stress levels for indoor corrosion testing can be determined through numerical algorithms, ensuring the constraints of the acceleration factor and environmental stress limits are met.

### 4.3. Case Study and Validation

In this case study, we estimate the parameters of the corrosion degradation model and the link function for LY12CZ aluminum alloy using data from field corrosion exposure tests documented in previous research [44]. To evaluate the impact of various environmental factors thoroughly and impartially on the corrosion rate of LY12CZ aluminum alloy, we selected seven representative regions that span four distinct climatic zones, each with unique environmental characteristics. The multi-year average environmental parameters for these regions are detailed in Table 9. The referenced research applied a corrosion kinetics model to fit the weight loss data of LY12CZ specimens exposed to atmospheric conditions, resulting in parameters A and n, as shown in Table 10.

Given the different magnitudes of the environmental factors in each region, each factor was normalized by dividing by its maximum value across the seven locations before estimating the parameters of the link function model. The methods used to fit the link functions are the TR, GE, and GL models, as presented in detail in Section 3. The correlation coefficient (*R*^2^) is used as a basic index for model goodness-of-fit. The estimated parameters of the link function models are listed in Table 11.

For the link function model of parameter *A*, the two-step regression (TR) model exhibited a correlation coefficient (*R*^2^) of 0.749, whereas the General Eyring (GE) and General Log-Linear (GL) models had *R*^2^ values of 0.636 and 0.677, respectively. Among these models, the TR model most accurately associates parameter *A* with environmental variables.

For parameter *n*, the TR model also demonstrated the highest *R*^2^ value of 0.807, compared to 0.520 for the GE model and 0.735 for the GL model. This indicates that the TR model most effectively describes the relationship between parameter *n* and the environmental variables. Therefore, in the remainder of this case study, the TR model is used to predict the parameters for the indoor simulation environment.

For the parameters of the link function, a positive value indicates an increased effect, and a negative value indicates a decreased effect. Taking the results of the TR model as an example, $\gamma_{A1}{,\;\gamma }_{A2},\;{and\;\gamma }_{A3}$ are all higher than 0, revealing the accelerating corrosion effect of temperature, relative humidity, and chloride deposition. On the other hand, for $\gamma_{n1}{,\;\gamma }_{n2},\;{and\;\gamma }_{n3}$, the negative value means the corrosion process proceeds faster, and the long-term steady-state corrosion process occurs earlier when the three factors increase.

**Figure 3:**
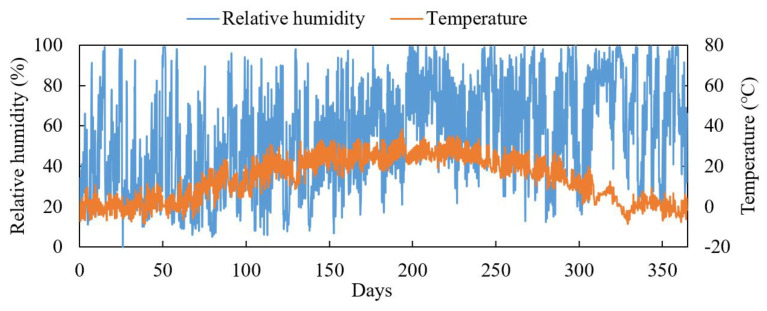
The observed environmental data for 2018.

A specific city in northern China is selected as the field corrosion environment. Temperature and relative humidity data for this location were obtained from the National Oceanic and Atmospheric Administration (NOAA) online database for the year 2018. Temperature and relative humidity readings were recorded every three hours, as illustrated in Figure 3. Due to the lack of long-term monitoring data for chloride ion deposition rates, an annual average value of 5 mg/d/m^2^ was used for the calculations.

**Figure 4:**
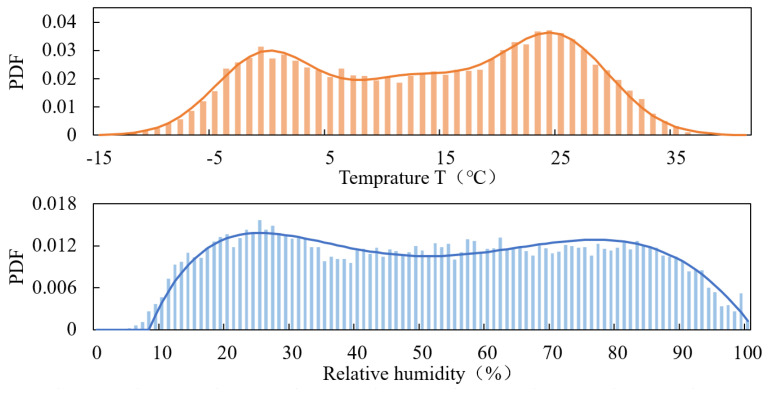
Histograms and PDF of environmental factors.

Analysis of the temperature distribution over one year revealed a clear bimodal pattern, leading to the use of a mixed normal distribution model to fit the temperature data, which provided an excellent fit, as shown in Figure 4. For relative humidity, a mixed beta distribution model was used to describe its distribution, as its values range from 0 to 1. The fitting results are presented in Figure 4. While the relative humidity fitting is not as precise as the temperature fitting—possibly due to the irregularity of rainfall events affecting humidity—the probability density functions for temperature and relative humidity were derived from these fittings.

$$f\left(T\right)=0.53f_{N}\left(1.16,3.69\right)+0.47f_{N}\left(24.8,3.52\right)$$

$$f\left(RH\right)=0.44f_{Be}\left(2.03,6.12\right)+0.56f_{Be}\left(3.98,2.06\right)$$

where $f_{N}\left(p1,\;p2\right)$ represents the probability density function (PDF) with the normal distribution of parameters p1 and p2, and $f_{Be}\left(p1,\;p2\right)$ represents the PDF with the beta distribution of parameters p1 and p2.

Then, the degradation parameters are modified according to Equation (39). As the lowest temperature is lower than 0 °C, corrosion reactions will halt. Thus, *T_L_* = 0 in this case, and then

$$A^{\prime }=\int_{{RH}_{L}}^{{RH}_{U}}\int_{0}^{T_{U}}\xi_{A}\cdot f\left(T\right)\cdot f\left(RH\right)dT\;dRH=0.891$$

$$n^{\prime }=\int_{{RH}_{L}}^{{RH}_{U}}\int_{0}^{T_{U}}\xi_{n}\cdot f\left(T\right)\cdot f\left(RH\right)dT\;dRH=0.354$$

Subsequently, we formulate the optimization problem for indoor corrosion testing of LY12CZ aluminum alloy. Assuming the temperature and humidity ranges applicable in the laboratory are 20–50 °C and 60–99%, respectively, the chloride ion deposition rate is controlled by applying 0.3 µL/cm^2^ of 0.1 mol/L NaCl solution to the surface of the samples using a microinjector. The solution is evenly spread and then dried before placing the samples in the test chamber. This process is repeated every 24 h to maintain a consistent chloride deposition rate of 35.1 mg/m^2^/day. Given these conditions, the optimization problem is structured as follows:
Minimize$\Psi \left(\zeta \right)={\left(\xi_{n}\left(\vec{S}\right)-0.354\right)}^{2}$subject to${\left(\frac{\xi_{A}\left(\vec{S}\right)}{0.891}\right)}^{\frac{1}{0.354}}=k$20≤T≤5060≤RH≤99

**Figure 5:**
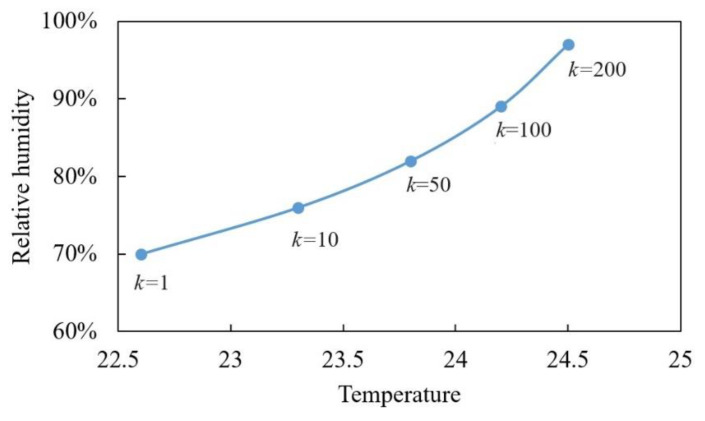
Environmental factors vs. acceleration factor.

The nonlinear optimization problem is solved using numerical methods, and the optimal solution for CMed in indoor corrosion testing is presented in Table 12 and Figure 5. It is observed that both temperature and humidity stress levels increase with the acceleration factor *k*. However, the range of temperature variation is relatively small due to the high sensitivity of CMed to temperature; a slight increase in temperature rapidly alters CMed. Consequently, increasing the acceleration factor *k* necessitates a significant change in humidity to maintain a consistent CMed.

### 4.4. Discussion

This paper aims to provide a framework of the optimal design process of an accelerated corrosion test that has the highest efficiency under the condition of consistent corrosion mechanisms. There are three steps: corrosion mechanism equivalence evaluation, link function construction and parameter estimation, and corrosion test design optimizations.

In the first step, a corrosion test both in laboratory and field environments is needed to validate the consistency of the corrosion mechanism. This step can determine the best laboratory environment that can simulate the field environment with highest CMed. In the next step, link function is built to correlate the environment factors with corrosion parameters, accounting for the actual influence of the field dynamic environment on the corrosion process. In the last step, the optimization problem is formulated subject to the constraints of mechanism equivalence and environment distribution range.

This paper is the first one to propose the framework of optimal corrosion experiment design. No similar methods are found in published papers. There are references that can be used as examples and help to understand the process of mechanism equivalence evaluation [31,45] and link function construction [46,47]. These refences studied different materials in different environments, including metals [16,17] and coatings [8,48] in both laboratory and field environments.

It is noted that there are potential sources of error and variability in the data in every step of the proposed method. Corrosion data variation may be induced by scatter in material difference and unknown measurement errors, while the model structure of the link function may cause bias of the underlying relationship between corrosion parameters and environment factors. Additionally, the dynamic field environment has periodic pattern and random fluctuation, which will lead to inevitable uncertainty of the environment data and models. All these sources can have different impacts on the performance of the proposed method, which one must pay attention to in practical applications.

## 5. Conclusions

This study presents a novel method for designing indoor accelerated corrosion tests with equivalent degradation mechanisms compared to field tests. The main innovation of the proposed indoor accelerated corrosion test design method is its basis in the acceleration factor invariant principle to derive mechanism-related parameters in the corrosion model. Then, a modified link function is proposed to account for the impact of dynamic environments on traditional link functions. Finally, an optimization objective function, maximizing the Corrosion Mechanism Equivalence Degree (CMed), is formulated, incorporating environmental stress limits and acceleration factors as constraints of the optimization problem. This approach allows for the optimal experimental design that maintains equivalent corrosion mechanisms between indoor and field tests. This method can provide more accurate information for product life prediction, health management, material selection, maintenance decisions, task re-planning, and so on.

## Figures and Tables

**Table 1:** Notations used in this paper.

A, n	Parameters of the corrosion model.
C	Corrosion loss, such as corrosion mass loss, corrosion depth, etc.
CMed	Corrosion Mechanism Equivalence Degree.
$\vec{E}$	Vectors of average environmental factors.
$f\left(\cdot \right)$	The probability density function of the environmental factor.
GE	General Eyring model.
GL	General Log-Linear model.
K	Acceleration corrosion factor.
$\vec{\gamma }$	Coefficient vectors of environmental factors.
$s_{i}$	Indoor accelerated corrosion environment.
$s_{f}$	Field corrosion environment.
$t_{i}$	Indoor corrosion time.
$t_{f}$	Field corrosion time.
TR	Two-step regression model.
Z	Impedance modulus of the coating at low frequencies.
α, β	Parameters of the coating impedance degradation model.
$\xi_{\theta }\left(\cdot \right)$	The optimal link function describing the effect of environmental factors with parameter θ.
η, λ	Parameters of the coating gloss-aging model.
ΔG	The gloss loss of the coating.

**Table 2:** Chemical composition (wt%) of 7B04 aluminum alloy.

Element	Zn	Mg	Cu	Ni	Ti	Cr	Mn	Fe	Si	Al
Content	6.09	2.54	1.65	<0.05	0.017	0.13	0.26	0.14	0.049	Bal.

**Table 3:** Average atmospheric environment data of test stations.

Test Station	Longitude	Latitude	Altitude (m)	Temperature (°C)	RH (%)	SO_2_ (mg/m^3^)	Cl^−^ (mg/m^3^)	pH of Rainfall
Jiangjin	106°15′ E	29°19′ N	208.6	17.9	81	1.018	0.007	4.3
Wanning	110°05′ E	18°58′ N	12.3	25.0	85.4	0.082	0.6099	5.4

**Table 4:** The test parameters for SO_2_/salt spray combined cyclic test.

Test Condition	Value
Immersion solution	5% NaCl
Immersion temperature	48 °C
Ambient temperature	35 °C
SO_2_ gas flow rate	10 cm^2^/(min·m^3^)
pH	2.5–3.2

**Table 5:** Corrosion degradation model parameters in different corrosion environments.

Corrosion Test	A	n	Goodness-of-Fit
Jiangjin	0.9566	0.2595	0.9729
Wanning	6.4045	0.7961	0.9848
Salt spray	4.1954	0.5313	0.8665
Cyclic immersion	5.4303	0.4472	0.9630
SO_2_/salt spray	0.1172	0.7966	0.8989

Note: The unit of field test time is year, and the unit of indoor test time is h.

**Table 6:** CMeds of 7B04 in different corrosion environments.

	Jiangjin	Wanning
Salt spray	−0.0474	0.6775
Cyclic immersion	0.2767	0.5617
SO_2_/salt spray	−1.0697	**0.9994**

**Table 7:** Averaged stress levels used in each accelerated aging chamber.

Chamber	Temperature (K)	UV Radiation (W/m^2^nm)	SO_2_ Aerosol *
1	310.7	0.58	1
2	311.2	0.41	0
3	330.7	0.42	1
4	331.4	0.57	0
5	326.9	0.60	1
6	327.4	0.43	1
7	329.4	0.63	0
8	329.6	0.62	1

* 1—SO_2_ aerosol application 40 min once a week; 0—no SO_2_ aerosol application.

**Table 8:** Model parameters and CMeds of gloss degradation in different environments.

Corrosion Test	η	λ	Goodness-of-Fit	CMed
Field	0.1175	0.0312	0.9779	—
Chamber 1	0.1373	0.1116	0.9887	0.8315
Chamber 2	0.0891	0.0785	0.9577	0.7583
Chamber 3	0.0231	0.4474	0.9726	0.1966
Chamber 4	0.0442	0.2395	0.9826	0.3762
Chamber 5	0.1000	0.2780	0.9894	0.8511
Chamber 6	0.0826	0.2863	0.9865	0.7029
Chamber 7	0.1036	0.1713	0.9957	**0.8817**
Chamber 8	0.0692	0.3495	0.9942	0.5889

**Table 9:** Averaged environmental parameters of typical regions [44].

Region	T (°C)	RH (%)	Cl^−^ (mg/d/m^2^)
Qingdao	12.5	71	24.98
Jiangjin	18.4	81	0.67
Wanning	24.6	86	43.53
Qionghai	24.5	86	19.88
Guangzhou	22.4	78	2.35
Beijing	12.0	57	0.49
Wuhan	16.9	77	1.05

**Table 10:** Corrosion degradation model parameter estimation of LY12CZ in typical regions.

	Qingdao	Jiangjin	Wanning	Qionghai	Guangzhou	Beijing	Wuhan
A	7.08	4.06	4.18	0.88	0.80	0.44	0.48
n	0.47	1.01	0.06	0.86	0.97	0.86	0.67

**Table 11:** Parameters of the link function models.

Parameter	TR	GE	GL
$\gamma_{A0}$	**−205.04**	5.02 × 10^−10^	−483.97
$\gamma_{A1}$	**54,552.49**	−6295.90	1.11 × 10^5^
$\gamma_{A2}$	**25.16**	0.24	98.46
$\gamma_{A3}$	**0.098**	0.42	0.083
Goodness-of-fit	**0.749**	0.636	0.677
$\gamma_{n0}$	**6.00**	307.42	11.25
$\gamma_{n1}$	**−1450.52**	1687.10	−3168.80
$\gamma_{n2}$	**−0.094**	0.19	−0.56
$\gamma_{n3}$	**−0.019**	−0.21	−0.029
Goodness-of-fit	**0.807**	0.520	0.735

**Table 12:** CMed-optimality test scheme under different acceleration factors.

	k=1	k=10	k=50	k=100	k=200
T	22.6 °C	23.3 °C	23.8 °C	24.2 °C	24.5 °C
RH	70%	76%	83%	88%	97%

## Data Availability

The original contributions presented in the study are included in the article, further inquiries can be directed to the corresponding author.
